# Corncobs as a Potential Source of Functional Chemicals

**DOI:** 10.3390/molecules181113823

**Published:** 2013-11-08

**Authors:** Ahmed Ashour, Mohamed Amer, Amani Marzouk, Kuniyoshi Shimizu, Ryuichiro Kondo, Saleh El-Sharkawy

**Affiliations:** 1 Department of Agro-environmental Sciences, Faculty of Agriculture, Kyushu University, 6-10-1 Hakozaki, Higashi-ku, Fukuoka 812-8581, Japan; 2 Department of Pharmacognosy, Faculty of Pharmacy, Mansoura University, Mansoura 35516, Egypt; 3 Department of Pharmacognosy, Faculty of Pharmacy, Delta University for Science and Technology, Mansoura 35516, Egypt

**Keywords:** matairesinol derivative, corncobs, agricultural waste

## Abstract

Phytochemical examination of corncob extracts led to the isolation of a new lignan identified as 7,7'-dihydroxy-3'-*O*-demethyl-4-methoxymatairesinol, together with seven known compounds, identified as *β*-sitosterol, *β*-sitosteryl-β-d-glucoside, 6*β*-hydroxy-campest-4-en-3-one, 5α,8α-epidioxyergosta-6,22-dien-3β-ol, tricin, kaempferol and *p*-coumaric acid. The isolated compounds were identified by one and two-dimensional NMR spectroscopies and mass spectrometry.

## 1. Introduction

Corncobs are an important byproduct of the sweet corn processing industry in Egypt, where they represent about 15% of the total corn production and the total volume of this by-product generated from the total volume of corn was estimated to be 54,424 ton in 2008 (personal communication, Egyptian Directorate of Agriculture). Worldwide, corncobs are either used as animal feed or returned to the harvested field as fertilizer [1].

Corncobs contain approximately 39.1% cellulose, 42.1% hemicellulose, 9.1% lignin, 1.7% protein, and 1.2% ash [2]. Due to their chemical composition, corn residues show great potential as a renewable raw material for producing a variety of added-value chemicals, such as lactic acid, citric acid, sugars, and ethanol [3,4,5,6]. On the other hand, the secondary metabolites and constituents of corncobs remain unclear. Development of an efficient way to utilize corncobs will require additional research into the chemical nature of this environmental agro-waste and its potential application to the production of valuable chemicals and pharmaceuticals.

## 2. Results and Discussion

**Figure 1:**
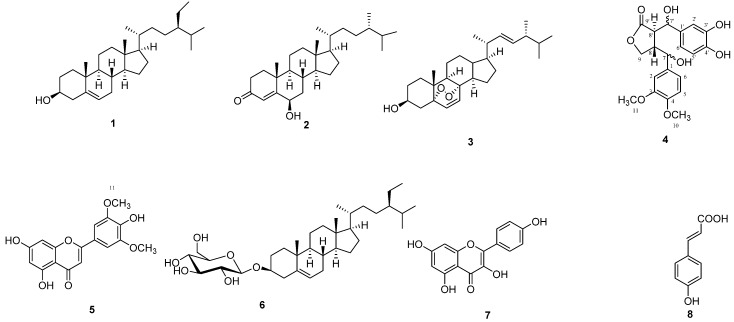
Chemical structures of isolated compounds.

In this work a new lignan, 7,7'-dihydroxy-3'-*O*-demethyl-4-methoxymatairesinol (**4**), was isolated from corncobs along with seven known compounds (Figure 1).

The known compounds were identified by comparing their physical, chemical and spectral data to those listed in the literature as follows:

*β-*Sitosterol (**1**) was identified by m.p., I.R. as well as co-chromatography with an authentic sample. Some epidemiological results have indicated that *β*-sitosterol has a protective role against some cancers such as human colon cancer [7], human prostate cancer [8] and human breast cancer [9].

6*β*-Hydroxycampest-4-en-3-one (**2**) was identified by ^1^H-NMR, ^13^C-NMR and LC-MS. This compound has never been isolated from corncobs or from any members of the family Poaceae. The data was compared with the published data [10,11]. Some results show that derivatives of campesterol exhibit significant cytotoxicity against NSCLC-N6 lung cancer cell lines [11].

5α,8α-Epidioxyergosta-6,22-dien-3β-ol (**3**) was identified by ^1^H-NMR and ^13^C-NMR. This is the first report of the isolation of **3** from the family Poaceae. This compound was previously isolated from the fungi *Bovistella radicata*, family Lycoperdaceae [12], the fungus *Daedalea quercina* and *confragosa* var. *tricolor* [13], and from the Chinese soft coral *Sinularia flexibilis* [14]. The data were further confirmed by HMBC experiments, as well as by comparison with published data [13]. Published results [15] showed that ergosterol inhibited the Matrigel-induced neovascularization, suggesting that compound **3** as a derivative of ergosterol might have beneficial effects.

The new lignan derivative 7,7'-dihydroxy-3'-*O*-demethyl-4-methoxy matairesinol (**4**) was isolated as a yellowish brown amphorous powder. Its molecular formula was established as C_20_H_22_O_8_ by EIMS (*m/z* 390, [M]^+^). The presence of a phenolic dibenzylbutyrolactone lignan skeleton was suggested by the UV spectrum, which showed absorption maxima at 236 and 279 nm and a bathochromic shift upon addition of alkali, along with a γ-lactone carbonyl absorption at 1733 cm^−1^, a hydroxyl group absorption at 3409 cm^−1^ and aromatic ring absorptions at 1608, 1517 cm^−1^ in the IR spectrum [16].

The ^1^H-NMR spectrum showed the presence of aromatic proton signals at δ 6.77 to 6.89 integrated for six protons that matched the ^13^C data, which indicated the presence of six aromatic protonated carbons at δ 108.1 (C-2), 107.8 (C-5), 118.1 (C-6), 114.7 (C-2'), 114.5 (C-5'), 118.4 (C-6'). The singlet at δ 3.89 integrated for six protons, suggesting the presence of two methoxy groups and consistent with the ^13^C peak appearing at δ 56.2. Other signals in the ^1^H-NMR at δ 4.32 [1H, *dd*, *J* = 9.5 Hz (9a, 9b), 9.3 Hz (9a, 8)] and 4.01 [1H, *dd*, *J* = 9.5 Hz (9b, 9a), 9.1 Hz (9b, 8)] were assigned to H-9a and H-9b, respectively. The proton signal at δ 3.44 that integrated for two protons was assigned to H-8 and H-8'.

The stereochemistry at C-7 and C-7' is not described, since it was not possible to make a safe assignment with only one diastereoisomer being isolated. The full assignment and connectivities of other positions was confirmed through HMQC and HMBC experiments.

The HMQC-spectrum revealed the correlation of the proton signal at δ 5.33 that integrated for two protons with the carbon signals at δ 84.7 (C-7) and 83.4 (C-7'). Both the proton signals at δ 4.01 (H-9b) and that at 4.32 (H-9a) were correlated with the carbon signal at δ 72.2 (C-9), and the proton signals at δ 3.89 were correlated with the carbons at δ 56.2 (C-10 and C-11). The signal at δ 3.44 integrating for two protons was correlated with the carbon signals at δ 56.1 (C-8') and 53.4 (C-8).

HMBC experiment data was as follows, with several long-range couplings being observed between the key protons and their neighboring carbons. The proton signal at δ 5.33 (H-7) showed correlations with the carbon signals at δ 72.7 (C-9), 118.1 (C-6) and 108.1 (C-2). The proton signal at δ 5.33 (H-7') showed correlations with the carbon signals at δ 177.1 (C-9') and 118.4 (C-6'). The proton signal at δ 4.01 (H-9b) showed a correlation with the carbon signal at δ 56.1 (C-8'), while the proton signal at δ 4.32 (H-9a) showed a correlation with the carbon signal at δ 84.7 (C-7).The proton signal at δ 6.77 (H-2 and H-6) showed correlations with the carbon signals at δ 84.7 (C-7), 107.8 (C-5), 108.1 (C-2), 118.1 (C-6), 146.8 (C-4) and 147.0 (C-3). The proton signal at δ 3.89 (H-10 and H-11) showed correlations with the carbon signals at δ 146.8 (C-4) for H-10 and the carbon signal at δ 147.0 (C-3) for H-11, which confirmed the position of the methoxy groups. The proton signal at δ 3.44 (H-8) showed a correlation with the carbon signal at δ 132.3 (C-1).

The EIMS spectrum showed a molecular ion peak at *m/z* 390 and fragment ions at *m/z* 372 (69%) [M−H_2_O]^+^ and 151 (100%) [M−H_2_O−C_11_H_9_O_5_]^+^, which confirmed the structure of the lignan. It is also worth mentioning that the presence of a base peak at 151 further confirmed the position of the *ortho*- methoxy groups in the ring [17].

Finally, from the above physical, chemical and spectroscopic data and through comparison with the data published in the literature [18], compound **4** could be identified as 7,7'-dihydroxy-3'-*O*-demethyl-4-methoxymatairesinol. This compound is a new compound which has not been isolated from any natural source before. It belongs to the lignans, which are a large class of secondary metabolites in plants, with numerous biological effects in mammals, including antitumor and antioxidant activities [19].

Tricin (**5**) was identified by UV, I.R., ^1^H-NMR, and ^13^C-NMR. Its identity was confirmed by comparison of its spectroscopic data with the published data for the compound [20]. It is worth mentioning that this compound was previously isolated from the family Poaceae, but this is the first report of its isolation from corncobs. Results support that tricin might be beneficial in hepatic stellate cells targeting therapeutic or chemopreventive applications for hepatic fibrosis [21] as well as for its antioxidant effects [22].

*β*-Sitosteryl-β-d-glucoside (**6**) was identified by m.p., I.R. and co-chromatography with an authentic sample. Results showed that *β*-sitosteryl-β-d-glucoside may be a useful candidate for the development of new drugs to treat endotoxemia and inflammation accompanied by the overproduction of nitric oxide, as it reduced nitric oxide production from lipopolysaccharides-induced RAW 264.7 cells. In addition, it strongly inhibited the interleukin 6 (IL-6) activities of stimulated macrophages [23].

Kaempferol (**7**) was identified by UV, I.R., ^1^H-NMR, ^13^C-NMR. Its identity was confirmed by comparison of its spectroscopic data with the published data for kaempferol [20]. It is worth mentioning that this compound was isolated before from the family Poaceae, but this is the first report of its isolation from corncobs. Previous results support that kaempferol has a depigmenting, anti-inflammatory activity [24] and antioxidant activity [22].

*p*-Coumaric acid (8) was identified by ^1^H-NMR and ^13^C-NMR. The identity of the structure was further confirmed by co-chromatography with an authentic sample. It is worth mentioning that this compound was isolated from corncobs for the first time in this work. Some epidemiological results supported that *p*-coumaric acid is a potent inhibitors of 5-*S*-cysteinyldopamine-induced neurotoxicity [25]. Results also showed that hydroxycinnamic acids possess numerous biological effects, including antioxidant, antiallergic, antimicrobial, and immunomodulatory activities [26].

## 3. Experimental

### 3.1. General

Column chromatography: Silica gel (70–230 mesh) (Merck, Darmstadt, Germany), cartridge Rp 18 (Merck), and silica gel 60 F254 precoated aluminum sheets were used for the TLC. IR spectra were recorded on a Perkin-Elmer 1430 ratio recording spectrophotometer (Perkin Elmer, Waltham, MA, USA). Ultraviolet spectral data for the isolated compounds was performed on a UV/Visible spectrophotometer (Shimadzu 1601 PC, model TCC-240 A; Shimadzu, Kyoto, Japan). Melting points were determined on a Fisher-Johns Scientific Co. (Waltham, MA, USA) melting point apparatus, USA. EIMS was carried out on (JEOL JMS600 spectrometer, Fukuoka, Japan) while LCMS was conducted using a 3200 Q-trap LC/MS/MS system (Applied Biosystems, Foster City, CA, USA). The software used to control this equipment and to acquire and process data was Analyst version 1.4.1 (MDS Sciex, Toronto, ON, Canada). The analytes were ionized using an electro-spray ionization (ESI) interface operated in positive mode. The analysis was conducted using Q1 scan and the mass scan range was *m/z* 50–500 (0.15 s/scan). The ^1^H-, ^13^C-, APT, HMBC and HMQC NMR spectra were analyzed on JEOL JNM ECA instruments at 400, 500 MHz for ^1^H- and 100, 125 MHz for ^13^C-NMR spectra.

Authentic samples of *β*-sitosterol, *β*-sitosterol glucoside and *p*-coumaric acid were obtained from previously isolated and identified samples in the Department of Pharmacognosy, Faculty of Pharmacy, Mansoura University.

### 3.2. Agro-Waste Material

Corncobs were obtained on August 2008 by Prof. Dr. Ahmed Nader, Agronomy Department, Faculty of Agriculture, Mansoura University, from plants grown in a field at the university campus. Corncobs type was identified as *Zea mays* hybrid individual 3080. They were milled to a particle size of 1.25 μm. A voucher specimen (No. 1536) was deposited at the Department of Pharmacognosy, Faculty of Pharmacy, Mansoura University.

### 3.3. Extraction and Isolation Procedures

Six kilograms of the powdered corncobs were extracted by cold maceration with MeOH (6 × 10L). The combined methanolic extracts were concentrated to a syrupy consistency under reduced pressure at 40 °C and then allowed to dry in a desiccator over anhydrous CaCl_2_ to a constant weight (184.5 g). The dried methanolic extract was dissolved in 200 mL MeOH, diluted with the same volume of distilled water and extracted successively with petroleum ether (5 × 1 L), CH_2_Cl_2_ (5 × 1 L) and EtOAc (5 × 1 L). The extracts, in each case, were evaporated to dryness under reduced pressure at 40 °C to yield the petroleum ether fraction (fraction A, 27 g, 0.45%), CH_2_Cl_2_ fraction (fraction B, 38 g, 0.63%) and EtOAc fraction (fraction C, 7 g, 0.12%).

Fraction A (27 g) was chromatographed on a silica gel column (62.5 × 4.5 cm) and eluted with mixtures of ethyl acetate in petroleum ether (0%–30% v/v). Fractions of 100 mL were collected and monitored by TLC using ethyl acetate/petroleum ether (5%, 8%, 10%, 15%, 20%, 25%, 30% and 40%) as developing systems and vanillin/H_2_SO_4_ acid as a spray reagent. Similar fractions were pooled to yield three subfractions: A1 (3.6 g), A2 (50 mg) and A3 (100 mg). Fraction A1 was purified by repeated crystallization from 20% chloroform/methanol to yield compound **1** (2.8 g). Fraction A2 was purified by repeated crystallization from methanol to afford compound **2** (13 mg). Fraction A3 was purified by rechromatography on a silica gel column (60 × 1.0 cm) and eluted with ethyl acetate in petroleum ether (10%–20% v/v) followed by further purification on a cartridge of reversed phase silica RP18 isocratically eluted with MeOH–Water (90:10) to give rosette crystals of compound **3** (12 mg).

Fraction B (38 g) was chromatographed on a silica gel column (65 × 4.5 cm) and eluted with different proportions (5%–100%) of ethyl acetate in a mixture of petroleum ether/methylene chloride (50:50). Fractions of 100 mL were collected and monitored by TLC using ethyl acetate/petroleum ether (20%–90%) as developing systems and vanillin/H_2_SO_4_ acid as a spray reagent followed by heating at 110 °C for 1 min. Similar fractions were pooled to yield three subfractions, B1 (390 mg), B2 (300 mg) and B3 (250 mg). Fraction B1 was purified by chromatography on a silica gel column (60 × 1.0 cm) and eluted with ethyl acetate in petroleum ether (20%–25% v/v) followed by purification by re-chromatography on a silica column (40 × 1 cm) and eluted isocratically with methylene chloride to give an amphorous powder of compound **4** (26.8 mg). Fraction B2 was purified by repeated crystallization from methanol to afford compound **5** (200 mg). Fraction B3 was also purified by repeated crystallization from 10% hot dichloromethane–methanol to afford fine needles of compound **6** (150 mg).

Fraction C (7 g) was chromatographed on a silica gel column (100 × 1.5 cm) and eluted with ethyl acetate in petroleum ether (50%–100%), and then the elution was continued using methanol/ethyl acetate (20%–50%) to give two fractions, C1 (20 mg) and C2 (25 mg). These fractions were purified by repeated crystallization from methanol to yield compound **7** (12 mg) and compound **8** (15 mg), respectively.

### 3.4. Identification of the Isolated Compounds

*β-Sitosterol* (**1**). White needles, m.p. 138–142 °C, TLC (silica gel GF_254_ and petroleum ether/EtOAc (90:10 v/v) as developer): R*_f_* = 0.22; violet color when sprayed with vanillin/sulfuric acid spray reagent and heating at 110 °C for 1 minute. IR (KBr, υ_max_): 3414, 2935, 2866, 1633, 1461 and 1377 cm^−1^.

*6β-Hydroxycampest-4-en-3-one* (**2**). White crystals. On developed TLC plates, it quenched UV_254_ light and gave a yellow color after heating with vanillin/H_2_SO_4_ spray reagent. It had an R*_f_* value of 0.37 with petroleum ether/EtOAc (70:30 v/v) as developer. LC-MS 414 (M^+^), ^1^H and ^13^C-NMR data are comparable to the published data [10,11].

*5α,8α-Epidioxyergosta-6,22-dien-3β-ol* (**3**). Colorless rosette crystals. On precoated GF_254_ silica gel plates, it showed an R*_f_* value of 0.29 using 30% ethyl acetate/petroleum ether as eluent. ^1^H and ^13^C-NMR data are comparable to the published data [13].

*7,7'-Dihydroxy-3'-O-demethyl-4-methoxymatairesinol* (**4**). Yellowish brown amphorous powder. On developed TLC plates, it has an R*_f_* value of 0.28 using 50% ethyl acetate/petroleum ether as developer. EIMS (*m/z* 390, [M]^+^). UV λ_max_ nm (in MeOH): 236 and 279. IR (KBr, υ_max_): 3409, 2925, 1733, 1608 and 1517 cm^−1^, ^1^H-NMR (CDCl_3_, 400 MHz, δ ppm): 6.77 (m, H-2), 6.89 (m, H-5), 6.77 (m, H-6), 5.33 (*d*, *J* = 6.9 Hz, H-7), 3.44 (m, H-8), 4.01(*dd*, *J* = 9.5, 9.1 Hz, H-9b), 4.32 (*dd*, *J* = 9.5, 9.3 Hz, H-9a), 3.89 (s, H-10), 3.89 (s, H-11), 6.88 (m, H-2'), 6.88 (m, H-5'), 6.79 (m, H-6'), 5.33 (*d*, *J* = 7.4 Hz, H-7'), 3.44 (m, H-8'). ^13^C-NMR (CDCl_3_, 100 MHz, δ ppm): 132.3 (C-1), 108.1 (C-2), 147.0 (C-3), 146.8 (C-4), 107.8 (C-5), 118.1 (C-6), 84.7 (C-7), 53.4 (C-8), 72.7 (C-9), 56.2 (C-10), 56.2 (C-11), 131.1 (C-1'), 114.7 (C-2'), 145.3 (C-3'), 146.1 (C-4'), 114.5 (C-5'), 118.4 (C-6'), 83.4 (C-7'), 56.1 (C-8'), 177.1 (C-9').

*Tricin* (**5**). Yellow needles, UV λ_max_ nm (in MeOH): 350 and 262. On developed TLC plates, it showed an R*_f_* value of 0.45 in 5% methanol/methylene chloride. IR (KBr, υ_max_): 3330, 1733, 1614, 1506 cm^‑1^, ^1^H and ^13^C-NMR data are comparable to the published data [20].

*β-Sitosteryl-β-D-glucoside* (**6**). White amorphous powder, m.p. 280–282 °C. It showed an R*_f_* value of 0.31 using 2% methanol/ethyl acetate as eluent system. IR (KBr, υ_max_): 3428, 2954, 2869, 1635, 1461, 1257, 1166, 1371, 1070 and 1022 cm^−1^.

*Kaempferol* (**7**). Yellow needles, m.p. 275–277 °C. It showed an R*_f_* value of 0.21 in 35% ethyl acetate/petroleum ether. UV λ_max_ nm (in MeOH): 368 and 267. ^1^H and ^13^C-NMR data are comparable to the published data [20].

*p-Coumaric acid* (**8**). White amorphous powder. On developed TLC plates, it had an R*_f_* value of 0.34 using 20% methanol/ethyl acetate. ^1^H-NMR (DMSO-*d*_6_, 500 MHz, δ ppm): 7.43 (*d*, *J* = 9.2 Hz, H-2), 6.79 (*d*, *J* = 9.2 Hz, H-3), 6.79 (*d*, *J* = 9.2 Hz, H-5), 7.43 (*d*, *J* = 9.2 Hz, H-6), 7.59 (*d*, *J* = 15.3 Hz, H-7), 6.27 (*d*, *J* = 15.3 Hz, H-8). ^13^C-NMR (DMSO-*d*_6_, 125 MHz, δ ppm): 125.9 (C-1), 129.8 (C-2), 115.5 (C-3), 159.8 (C-4), 115.5 (C-5), 129.8 (C-6), 145.3 (C-7), 114.3 (C-8), 169.8 (C-9).

## 4. Conclusions

Eight compounds have been isolated from corncobs. They are classified as two phenylpropanoids (one of them is a simple phenylpropanoid within the *p*-hydroxycinnamic acids subcategory which is *p*-coumaric acid and other is 7,7'-dihydroxy-3'-*O*-demethyl-4-methoxymatairesinol), two flavonoids (tricin and kaempferol) and four plant sterols (*β*-sitosterol, *β*-sitosteryl-β-d-glucoside, 6*β*-hydroxy campest-4-en-3-one and 5α,8α-epidioxyergosta-6,22-dien-3β-ol). These findings suggest corncobs as as potential source of interesting phytochemicals.
